# A Multicenter Real-World Study Evaluating the Hepatoprotective Effect of Polyene Phosphatidylcholine Against Chronic Hepatitis B

**DOI:** 10.3389/fmed.2022.842098

**Published:** 2022-06-22

**Authors:** Jinghang Xu, Yanan Fan, Yanyan Yu, Yifan Han, Qian Kang, Ning Tan, Yuqing Yang, Hongyu Chen, Jiali Pan, Xiaoyuan Xu

**Affiliations:** Department of Infectious Diseases, Peking University First Hospital, Beijing, China

**Keywords:** real world data (RWD), polyene phosphatidylcholine (PPC), magnesium isoglycyrrhizinate (IsoMag), glutathione (GSH), hepatitis B virus (HBV), chronic hepatitis B (CHB), alanine aminotransferase (ALT), aspartate aminotransferase (AST)

## Abstract

**Background:**

Polyene phosphatidylcholine (PPC) has been widely used to treat liver diseases in China. However, there is a lack of post-marketing evidence demonstrating its liver-protective efficiency among patients infected with hepatitis B virus (HBV). This study analyzed the multicenter real-world data to compare the effectiveness of PPC with those of magnesium isoglycyrrhizinate (IsoMag) and glutathione (GSH) in patients with liver injury.

**Methods:**

This study comprised the real-world data analysis of a multicenter, retrospective observational cohort. The data were retrieved from the Cooperative Registry of the Hospital Prescription in China between 1 October 2018, and 30 September 2019. A growth curve analysis was performed to compare the effects of different treatments on liver function longitudinally for up to 30 days after treatment commencement. In addition, the dose effect of the PPC treatment was investigated.

**Results:**

The final cohort included 6,052 patients with approximately 8% infected with HBV (*N* = 471). There were 1,649, 1,750, and 2,653 patients in the PPC, GSH, and IsoMag groups, respectively, with an average age of 53.9 years. In patients with HBV infection, the PPC treatment was associated with a significant decline in alanine aminotransferase (ALT) and aspartate aminotransferase (AST) levels (slopes: −3.7, 95% CI, −6.0 to −1.5 U/L/day; −2.4, 95% CI, −4.5 to −0.3 U/L/day, respectively). However, there were no significant differences in the effects among the three groups. In patients without HBV infection, the PPC treatment decreased ALT, AST, γ-glutamyl transferase (GGT), and albumin levels (−5.2, 95% CI, −5.8 to −4.5 U/L/day; −3.5, 95% CI, −4.2 to −2.7 U/L/day; −4.9, 95% CI, −6.2 to −3.7 U/L/day, −0.07, 95% CI, −0.09 to −0.04 g/L/day, respectively) and showed a stronger effect on lowering ALT levels than GSH (−2.6, 95% CI, −3.3 to −1.8 U/L/day, *p* < 0.05), as well as a stronger effect on lowering GGT levels than IsoMag (−1.4, 95% CI, −2.4 to −0.4 U/L/day, *p* < 0.05). PPC had no impact on prothrombin activity levels in patients with or without HBV infection. High-dose PPC exhibited a stronger effect on lowering ALT and AST levels than low-dose PPC.

**Conclusion:**

This was the first real-world multicenter study to demonstrate that PPC efficiently lowers ALT and AST levels in patients with liver diseases regardless of the status of HBV infection. PPC treatment showed a comparable or better effect compared with GSH and IsoMag treatments. High-dose PPC resulted in a stronger effect than low-dose PPC.

## Introduction

Hepatitis B virus (HBV) infection is a serious public health concern, especially in China, which has the largest HBV-infected population worldwide, with a 5–6% prevalence rate and 70 million HBsAg carriers in the country (1). HBV infection creates a tremendous financial burden for patients and the healthcare system. In 2016, the WHO developed the Global Health Sector Strategy on viral hepatitis, with the goal of eliminating hepatitis B by 2030 (2). The strategy is primarily targeted at the reduction of 90% of new infections and 65% mortality in 2030, using 2015 data as a reference.

Hepatitis B virus does not damage hepatocytes directly. The disease pathogenesis is closely related to abnormal adaptive immune responses induced by viral infections (3). The persistent infection and necroinflammation cause hepatocyte injury and can eventually lead to liver cirrhosis and even liver cancer. Chronic hepatitis B (CHB) is a lifelong disease that requires long-term treatment. In 2019, the Chinese Medical Association, Chinese Society of Infectious Disease, and Chinese Society of Hepatology updated the guidelines for the prevention and treatment of chronic hepatitis B (4) to comply with the WHO’s 2016 goal of eliminating viral hepatitis as a public health concern by 2030. The guideline lists clinical changes in CHB care that needs to be resolved and calls for a real-world study to evaluate the post-market safety, efficacy, and effectiveness of drugs used in the treatment of CHB in China. According to the guideline, the primary goal of treatment is to maximize the inhibition of viral replication, using anti-viral medications. Other treatments and therapeutic approaches are recommended to delay the progression of liver failure, improve the quality of life of patients, and increase life expectancy. Among those, anti-inflammatory, antioxidant, and hepatoprotective treatments can be used to reduce inflammatory liver damage. Antifibrosis treatment using Chinese medicine has also been reported.

Polyene phosphatidylcholine (PPC) is derived from soybean extract. It contains a 95%-pure mixture of major essential phospholipids (EPLs) that have a high affinity for cellular membranes. EPLs play key roles in repairing and regenerating liver cells (5). Studies have shown that PPC is effective in reversing hepatic damage by improving membrane-dependent hepatocellular function, exerting multiple effects, including anti-inflammation, antioxidant, antiapoptotic, and antifibrotic properties (6–9). Clinical trials showed that PPC treatment can benefit patients with various liver diseases, including non-alcoholic fatty liver disease, alcoholic liver disease, drug-induced liver injury, and even viral hepatitis. A double-blinded randomized controlled trial (10) by the U.S. Veterans Affairs reported that PPC significantly improved alanine aminotransferase (ALT) levels in hepatitis C virus (HCV)-infected patients with alcoholic liver disease. Another randomized trial also found that PPC treatment led to a higher rate of ALT response (>50% ALT-level decrease) compared with placebo in HCV-infected patients (11). In China, PPC injection has been used in the management of liver injury in patients with or without HBV infection for years (12). However, the post-marketing effectiveness of PPC has been rarely reported. In this study, we conducted the real-world data analysis using a large hospital registry in China, aiming to assess the effectiveness of PPC compared with those of magnesium isoglycyrrhizinate (IsoMag) and glutathione (GSH) in patients with liver injury.

## Materials and Methods

### Study Design

This was a multicenter, retrospective observational cohort study using registry data. The data were extracted from the Cooperative Registry of Hospital Prescription, a multicenter database, including clinical data from 264 Level-A hospitals in 16 provinces in China (13). The dataset included patient demographics, clinical indications, prescription and drug administration, and results of laboratory liver function tests. This study was approved by the Ethics Committee of Peking University First Hospital, and the need for patient informed consent was waived, owing to the retrospective nature of the study (No. 2021-434).

### Cohort Selection

**FIGURE 1:**
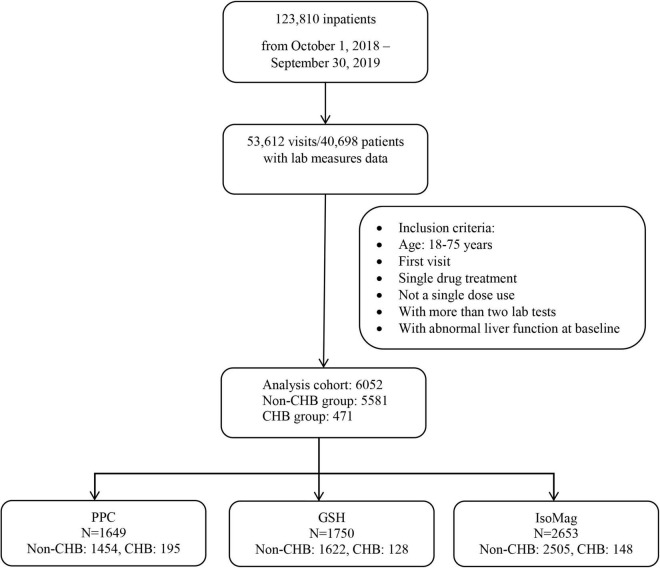
A flowchart of patient selection.

The study sample included patients with liver diseases who received one of the three drugs, IsoMag, GSH, or PPC, during hospitalization between 1 October 2018, and 30 September 2019. The eligibility criteria included ages between 18 and 75 years, abnormal ALT levels at admission, no concomitant use of other medications for liver disease, and available baseline and follow-up liver function tests within 30 days after treatment initiation. Liver function tests consisted of the following indicators: ALT, aspartate aminotransferase (AST), γ-glutamyl transferase (GGT), total bilirubin, albumin, and prothrombin activity (PTA). The patients were classified into two groups based on the type of liver disease, either the group with chronic hepatitis B infection or the group with other liver diseases, including non-alcoholic fatty liver disease, alcoholic hepatitis, hepatitis A, C, D, or E, cirrhosis, drug overdose, and liver cancer. Figure 1 displays the selection process of the analysis cohort.

### Statistical Analysis

Baseline characteristics of the patients were summarized using descriptive statistics based on the single hepatoprotective drug they received. The ANOVA or Kruskal–Wallis tests were used to compare baseline continuous variables between groups, and a Chi-square or Fisher’s exact test was used for categorical variables as appropriate. Growth curve analysis using a linear mixed-effects model was used to compare the effect of each drug on liver function up to 30 days post-treatment for patients with an HBV infection or other liver diseases (14). A random intercept was included to account for the within-subject correlations between repeated lab tests. A three-way interaction term of drug by time by HBV infection was used to compare the slopes of liver function parameters across treatments and HBV statuses. Age, sex, and hypertension were included as covariate adjustments. A subgroup analysis of PPC-treated patients was conducted to evaluate the differential effects of high-dose (no less than 930 mg PPC/day) and low-dose (less than 930 mg PPC/day) treatments. SAS 9.4 (Cary, NC, United States) was used for all statistical analyses. The significance level was set at *p* < 0.05, two-sided for the primary hypothesis test for which the slope was equal to zero. The Bonferroni method was used to correct for the multiple comparisons between treatment groups.

## Results

### Patient Characteristics

The registry included data from 123,810 hospitalized patients between 1 October 2018, and 30 September 2019. As shown in the flowchart (Figure 1), 6,052 patients were eligible for the analysis cohort. The average age was 53.9 ± 13.7 (mean ± SD) years. Overall, there were more men than women (62.5 vs. 37.5%, respectively). Approximately 8% of the patients (471 in total) had CHB (Table 1). The remaining 5,581 patients had other liver diseases. Table 1 shows that patients with CHB had similar ages to those of the non-CHB group, but a higher proportion of men (81.7 vs. 60.8%, respectively). The patients with CHB were more likely to receive PPC treatment compared to those without CHB (41.4 vs. 26.1%). The term of PPC treatment was similar between the two groups (11.9 ± 9.3 vs. 10.7 ± 8.5 days, CHB vs. non-CHB, respectively). Of note, the patients with CHB were more likely to receive lower PPC doses than those without CHB (49.2 vs. 40.9%). Table 2 compares patient characteristics grouped by the treatments. The final sample sizes were 1,649 patients in the PPC treatment group, 1,750 patients in the GSH treatment group, and 2,653 patients in the IsoMag group. The average ages of the PPC-treated patients were approximately 2 years lower than those of the patients in the other two groups. The average treatment durations varied from 9.4 to 10.8 days. The PPC treatment group had fewer patients with cancer, whereas the GSH group had more individuals with co-morbidities, such as diabetes and hypertension.

**TABLE 1:** Patients characteristics by non-CHB or CHB.

	Chronic hepatitis B			
	No (*N* = 5,581)	Yes (*N* = 471)	Total (*N* = 6,052)	*P*-value
**Age**				
Mean (SD)	54.0 (13.9)	53.3 (11.0)	53.9 (13.7)	0.17
Median (range)	56.0 (18.0–75.0)	54.0 (22.0–75.0)	56.0 (18.0–75.0)	0.004***
**Sex**				
F	2,186 (39.2%)	86 (18.3%)	2,272 (37.5%)	< 0.001***
M	3,395 (60.8%)	385 (81.7%)	3,780 (62.5%)	
**Treatment**				
GSH	1,622 (29.1%)	128 (27.2%)	1,750 (28.9%)	< 0.001***
IsoMag	2,505 (44.9%)	148 (31.4%)	2,653 (43.8%)	
PPC	1,454 (26.1%)	195 (41.4%)	1,649 (27.2%)	
**Duration of PPC treatment for PPC subgroup**				
Mean (SD)	10.7 (8.5)	11.9 (9.3)	10.8 (8.6)	0.08
**Cancer (hepatocellular carcinoma or other malignant tumors)**				
0	3,639 (65.2%)	424 (90.0%)	4,063 (67.1%)	< 0.001***
1	1,942 (34.8%)	47 (10.0%)	1,989 (32.9%)	
**Diabetes mellitus**				
0	5,016 (89.9%)	418 (88.7%)	5,434 (89.8%)	0.44
1	565 (10.1%)	53 (11.3%)	618 (10.2%)	
**Hypertension**				
0	4,475 (80.2%)	413 (87.7%)	4,888 (80.8%)	< 0.001***
1	1,106 (19.8%)	58 (12.3%)	1,164 (19.2%)	
**PPC dose**				
Low	595 (40.9%)	96 (49.2%)	691 (41.9%)	0.027**
High	859 (59.1%)	99 (50.8%)	958 (58.1%)	

CHB, chronic hepatitis B; F, female; M, male; GSH, glutathione; IsoMag, magnesium isoglycyrrhizinate; PPC, polyene phosphatidylcholine; SD, standard deviation. **p < 0.05, ***p < 0.001.

**TABLE 2:** Patients characteristics by treatment groups.

	Treatment				
	GSH (*N* = 1,750)	IsoMag (*N* = 2,653)	PPC (*N* = 1,649)	Total (*N* = 6,052)	*P-*value
**Age**					
Mean (SD)	54.5 (13.9)	54.7 (12.8)	52.1 (14.7)	53.9 (13.7)	< 0.001***
Median (range)	56.0 (18.0–75.0)	56.0 (18.0–75.0)	54.0 (18.0–75.0)	56.0 (18.0–75.0)	< 0.001***
**Sex**					
F	629 (35.9%)	1,078 (40.6%)	565 (34.3%)	2,272 (37.5%)	< 0.001***
M	1,121 (64.1%)	1,575 (59.4%)	1,084 (65.7%)	3,780 (62.5%)	
Level of hospital					
A	1,750 (100.0%)	2,653 (100.0%)	1,649 (100.0%)	6,052 (100.0%)	
**Treatment duration (days)**					
Mean (SD)	9.9 (7.0)	9.4 (7.9)	10.8 (8.6)		< 0.001***
Median (range)	9.0 (1.0–56.0)	7.0 (1.0–115.0)	9.0 (1.0–75.0)		< 0.001***
**Cancer (hepatocellular carcinoma or other malignant tumors)**					
0	1,204 (68.8%)	1,640 (61.8%)	1,219 (73.9%)	4,063 (67.1%)	< 0.001***
1	546 (31.2%)	1,013 (38.2%)	430 (26.1%)	1,989 (32.9%)	
**Diabetes mellitus**					
0	1,501 (85.8%)	2,424 (91.4%)	1,509 (91.5%)	5,434 (89.8%)	< 0.001***
1	249 (14.2%)	229 (08.6%)	140 (8.5%)	618 (10.2%)	
**Hypertension**					
0	1,307 (74.7%)	2,227 (83.9%)	1,354 (82.1%)	4,888 (80.8%)	< 0.001***
1	443 (25.3%)	426 (16.1%)	295 (17.9%)	1,164 (19.2%)	

GSH, glutathione; IsoMag, magnesium isoglycyrrhizinate; PPC, polyene phosphatidylcholine; SD, standard deviation.***p < 0.001.

Table 3 summarizes liver function results at the baseline based on the study groups. The levels of ALT, AST, and total bilirubin were all similar among the three groups. There was little difference in albumin level, although the *p*-value was significant. The patients in the GSH group had the highest PTA (102.5 ± 34.3%) and the lowest level of GGT [102.0 (33.0–1001.0) U/L].

**TABLE 3:** Baseline liver function test by treatment groups.

	Treatment				
	GSH (*N* = 1,750)	IsoMag (*N* = 2,653)	PPC (*N* = 1,649)	Total (*N* = 6,052)	*P*-value
**ALT (U/L)**					
Mean (SD)	107.3 (94.3)	125.9 (118.3)	133.2 (134.9)	123.1 (118.3)	0.047**
Median (range)	71.2 (28.6–797.5)	87.8 (28.2–740.0)	79.0 (29.0–730.0)	79.3 (28.2–797.5)	0.18
**AST (U/L)**					
Mean (SD)	116.7 (101.8)	113.8 (100.9)	120.2 (110.8)	116.4 (103.5)	0.72
Median (range)	80.0 (40.1–647.4)	73.8 (40.2–705.2)	77.0 (40.1–711.0)	77.0 (40.1–711.0)	0.63
**GGT (U/L)**					
Mean (SD)	157.2 (152.7)	204.3 (191.4)	209.0 (185.1)	193.2 (181.0)	< 0.001***
Median (range)	102.0 (33.0–1,001.0)	134.2 (32.2–977.3)	136.6 (34.2–988.7)	127.0 (32.2–1,001.0)	< 0.001***
**Total bilirubin (μ mol/L)**					
Mean (SD)	44.9 (69.8)	42.9 (68.1)	51.9 (83.6)	45.1 (71.2)	0.45
Median (range)	20.9 (3.1–421.9)	20.9 (3.7–430.7)	23.6 (2.6–436.9)	21.4 (2.6–436.9)	0.12
**PTA (%)**					
Mean (SD)	102.5 (34.3)	92.5 (26.7)	86.6 (23.4)	89.4 (26.0)	< 0.001***
Median (range)	110.0 (42.4 – 144.4)	91.0 (36.0 – 147.6)	90.7 (27.5 – 147.6)	91.0 (27.5 – 147.6)	0.005***
**Albumin (g/L)**					
Mean (SD)	35.1 (4.8)	34.4 (5.4)	35.0 (5.5)	34.8 (5.3)	< 0.001***
Median (range)	35.7 (16.2–55.4)	34.5 (10.1–54.2)	35.3 (5.4–50.0)	35.2 (5.4–55.4)	< 0.001***

GSH, glutathione; IsoMag, magnesium isoglycyrrhizinate; PPC, polyene phosphatidylcholine; ALT, alanine aminotransferase; AST, aspartate aminotransferase; GGT, γ-glutamyl transferase; PTA, prothrombin activity; SD, standard deviation. **p < 0.05, ***p < 0.001.

### Effects of Treatments on Liver Function Over Time

**FIGURE 2:**
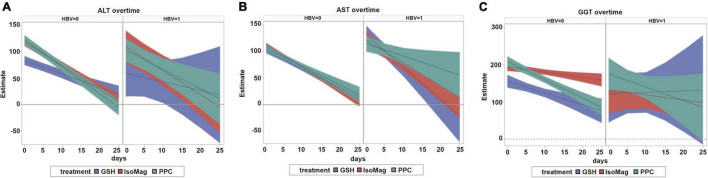
Predicted trajectories and 95% confidence bands. The left panel of each section corresponds to patients with non-CHB and the right panel corresponds to patients with CHB. **(A)** ALT over time. **(B)** AST over time. **(C)** GGT over time. CHB, chronic hepatitis B; GSH, glutathione; IsoMag, magnesium isoglycyrrhizinate; PPC, polyene phosphatidylcholine; ALT, alanine aminotransferase; AST, aspartate aminotransferase; GGT, γ-glutamyl transferase.

The results of the growth curve analyses are presented in Table 4, which compared the linear slopes of each variable according to the type of treatment. The fitted trajectories are shown in Figure 2.

**TABLE 4:** Compare slopes of liver function parameters by treatment groups between non-CHB and CHB patients.

	Non-CHB			CHB		
	GSH^1^	IsoMag^2^	PPC^3^	GSH^4^	IsoMag^5^	PPC^6^
ALT (U/L)	−2.6 (−3.3, −1.8)^3^	−4.5 (−5.1, −3.9)	−5.2 (−5.8, −4.5)^1^	−1.6 (−6.1, 2.8)	−4.6 (−6.8, −2.4)	−3.7 (−6.0, −1.5)
	*p* < 0.0001	*p* < 0.0001	*p* < 0.0001	*p* = 0.47	*p* < 0.0001	*p* = 0.001
AST (U/L)	−3.5 (−4.0, −3.0)	−4.2 (−4.7, −3.6)	−3.5 (−4.2, −2.7)	−6.2 (−8.5, −3.8)	−4.3 (−6.2, −2.4)	−2.4 (−4.5, −0.3)
	*p* < 0.0001	*p* < 0.0001	*p* < 0.0001	*p* < 0.0001	*p* < 0.0001	*p* < 0.0001
GGT (U/L)	−3.2 (−5.0, −1.4)	−1.4 (−2.4, −0.4)^3^	−4.9 (−6.2, −3.7)^2^	0.4 (−7.4, 8.3)	−1.4 (−6.0, 3.3)	−3.6 (−8.4, 1.3)
	*p* = 0.0005	*p* = 0.005	*p* < 0.0001	*p* = 0.91	*p* = 0.57	*p* = 0.15
Total bilirubin (μmol/L)	1.5 (1.0, 2.0)	0.6 (0, 1.3)	−0.7 (−1.8, 04)	1.3 (−0.8, 3.3)	0 (−2.7, 2.6)	5.4 (1.3, 9.5)
	*p* < 0.0001	*p* < 0.0001	*p* = 0.21	*p* = 0.23	*p* = 0.97	*p* = 0.01
PTA (%)	−1.9 (−2.6, −1.2)	−0.005 (−0.5, 0.5)	−0.1 (−0.3, 0.1)	−8.9 (−22.9, 5.0)	0.8 (−1.7, 3.3)	−0.3 (−1.1, 04)
	*p* < 0.0001	*p* = 0.86	*p* = 0.24	*p* = 0.21	*p* = 0.53	*p* = 0.35
Albumin (g/L)	0.01 (−0.05, 0.05)	0.002 (−0.02, 0.03)	−0.07 (−0.09, −0.04)	0.13 (−0.1, 0.35)	−0.23 (−0.37, −0.08)	0.05 (−0.14, 0.24)
	*p* = 0.85	*p* = 0.87	*p* < 0.0001	*p* = 0.27	*p* = 0.002	*p* = 0.60

*The superscript indicates the significant pair-wise comparison.CHB, chronic hepatitis B; GSH, glutathione; IsoMag, magnesium isoglycyrrhizinate; PPC, polyene phosphatidylcholine; ALT, alanine aminotransferase; AST, aspartate aminotransferase; GGT, γ-glutamyl transferase; PTA, prothrombin activity.*

Among the patients with CHB, the PPC treatment exhibited significant effects on reducing ALT (−3.7, 95% CI, −6.0 to −1.5 U/L/day) and AST (−2.4, 95% CI, −4.5 to −0.3 U/L/day) levels, whereas GSH and IsoMag showed considerably stronger effects on decreasing AST levels (Figure 2, right panel and Table 4, slopes −2.4 vs. −6.2 and −4.3 U/L/day, PPC vs. GSH and IsoMag, respectively), but the differences were not statistically significant. GSH did not affect other functional parameters, except for lowering AST. Meanwhile, the IsoMag group showed a decrease in albumin levels (*p* = 0.002).

In the patients with non-CHB, all three groups showed a significantly decreasing trend in ALT, AST, and GGT levels after the treatment (Figure 2, left panel). The PPC treatment showed a stronger effect on lowering ALT levels than GSH, as well as a stronger effect on lowering GGT levels than IsoMag. PPC did not affect total bilirubin levels, whereas GSH and IsoMag increased total bilirubin levels significantly. In addition, GSH lowered PTA (*p* < 0.0001). Only PPC treatment significantly lowered the albumin level, but the rate was quite small (−0.07 g/L/day) (Table 4).

When comparing the treatment effects among the patients with or without CHB, there were no significant differences in the effect of PPC on lowering ALT (−3.7, 95% CI, −6.0 to −1.5 vs. −5.2, 95% CI, −5.8 to −4.5 U/L/day, *p* = 0.22) and AST (−2.4, 95% CI, −4.5 to −0.3 vs. −3.5, 95% CI, −4.2 to −2.7, *p* = 0.33) levels. The PPC group showed increasing (slope: 5.4, 95% CI, 1.3 to 9.5 μmol/L/day, *p* = 0.01) and unchanged (*p* = 0.21) total bilirubin levels in the patients with CHB and the patients with non-CHB, respectively. PPC reduced GGT levels only in the patients with non-CHB (slope: −4.9, 95% CI: −6.2 to −3.7 U/L/day, *p* < 0.0001), but not in the patients with CHB (*p* = 0.15), and it had no effect on PTA in both patient groups. While it showed decreasing (slope: −0.07, 95% CI, −0.09 to −0.04 g/L/day, *p* < 0.0001) and unchanged (*p* = 0.60) albumin levels in the patients with non-CHB and the patients with CHB, respectively.

### Effect of Polyene Phosphatidylcholine Dosage

In China, the PPC injection comprises 232.5 mg PPC per vial. A high dose is defined as ≥4 vials/day, whereas a low dose is <4 vials/day. The three-way interaction of dose by time by HBV infection was not significant, indicating that the PPC dose effect was not modified by the HBV infection. Therefore, HBV was treated as a covariate in the subgroup analysis rather than a stratification factor.

**FIGURE 3:**
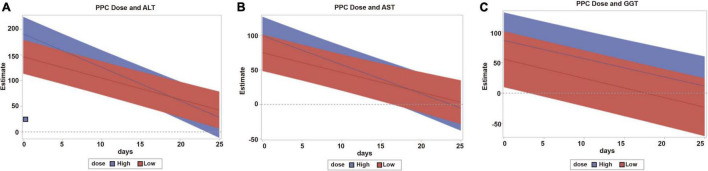
Comparison of the trajectories of high-dose and low-dose PPC treatment. **(A)** Dose effect on ALT change. **(B)** Dose effect on AST change. **(C)** Dose effect on GGT change. PPC, polyene phosphatidylcholine; ALT, alanine aminotransferase; AST, aspartate aminotransferase; GGT, γ-glutamyl transferase; high dose, 930 mg PPC/day; low dose, <930 mg PPC/day.

High-dose PPC demonstrated a significantly stronger effect on reducing ALT levels compared to that of low-dose PPC (−6.5 vs. −4.2 U/L/day, *p* = 0.001; Table 5). High-dose PPC also lowered AST levels faster than low-dose PPC, although the difference was at the borderline of statistical significance (−4.2 vs. −2.9 U/L/day, *p* = 0.05). The dosage did not affect GGT levels (*p* = 0.77; Figure 3).

**TABLE 5:** Compare effects of high vs. low dose PPC on liver function.

Slopes	Low dose <930 mg PPC/day	High dose ≥930 mg PPC/day	Difference
ALT (U/L)/day	−4.2 (−5.0, −3.3)	−6.5 (−7.6, −5.4)	−2.4 (−3.8, −0.9)
	*p* < 0.0001	*p* < 0.0001	*p* = 0.001
AST (U/L)/day	−2.9 (−3.9, −1.9)	− 4.2 (−5.2, −3.3)	1.3 (−2.7, 0.01)
	*p* < 0.0001	*p* < 0.0001	*p* = 0.05
GGT (U/L)/day	−3.2 (−3.8, −2.5)	−3.0 (−3.8, −2.1)	0.15 (−0.89, 1.29)
	*p* < 0.0001	*p* < 0.0001	*p* = 0.77

*PPC, polyene phosphatidylcholine; ALT, alanine aminotransferase; AST, aspartate aminotransferase; GGT, γ-glutamyl transferase.*

## Discussion

Real-world evidence (RWE) has drawn increasing attention recently. Searching real-world data or RWE on PubMed resulted in exponentially increasing numbers of publications. The information extracted from electronic health records, insurance claims and billing, data from registries and personal devices, and apps play an important role in healthcare (15, 16). This provides valuable data on randomized controlled trials.

Although PPC has been extensively used to treat patients with liver diseases in China for years, there is a lack of information about its effectiveness with respect to hepatoprotection in real-world clinical practice, especially for patients with CHB. This is the first study providing RWE on the effect of PPC on patients with CHB, using GSH and IsoMag as treatment controls, and using patients with non-CHB as patient population control.

Our results found that the PPC treatment effectively lowered ALT, AST, and GGT levels in patients with non-CHB liver diseases. The effectiveness was comparable with that of the GSH and IsoMag treatments, and it was even better at lowering the ALT level compared with GSH and lowering the GGT level compared with IsoMag. Furthermore, PPC was also effective in lowering ALT and AST levels in patients with CHB.

Oxidative stress plays a crucial role in liver damage. It alters DNA, proteins, and lipids in hepatocytes and is highly correlated with hepatic inflammation. Antioxidant medicines have exhibited efficacy in treating liver diseases (17). GSH is a major antioxidant in liver cells and has been proved to be efficacious in clinical use (18). PPC has a similar antioxidant effect that attenuates oxidative stress (8). This might, at least partially, explain the observed hepatoprotective effect of PPC in our study.

The hepatoprotective effect of PPC might also be related to the anti-inflammatory pathway. IsoMag is derived from the root of *Glycyrrhiza glabra*, which is a flowering herb in traditional Chinese medicine. Currently, it is an anti-inflammatory medicine widely used to treat liver diseases and has the potential of reversing the increase in ALT and AST levels (19, 20). Our results confirmed the hepatoprotective effect of IsoMag in patients both with and without HBV infection. Similar to those with IsoMag, PPC showed effects of lowering AST and ALT levels in both patient groups. However, only IsoMag treatments resulted in a significant decline in albumin levels in patients with CHB. This implies that their hepatoprotective mechanisms when treating patients with CHB might not be identical.

The PPC treatment significantly increased total bilirubin levels in patients with CHB, whereas GSH and IsoMag did so only in patients with non-CHB. Bilirubin is cleared by the liver, and elevated levels usually indicate a liver disorder. However, bilirubin is also a potent antioxidant (21). The role of elevated total bilirubin levels in the PPC-treated patients with CHB is unclear. Therefore, further investigation is warranted.

The high-dose PPC treatment lowered ALT levels faster than the low-dose treatment. Results also implied the same trend for AST levels. Therefore, if a patient is admitted with substantially high levels of ALT and AST, high-dose administration might be a better option to control the ALT and AST levels faster.

Although GSH and IsoMag mainly served as drug controls for the PPC treatment, we found that both drugs significantly increased the total bilirubin level in the patients without CHB, indicating a risk of potential liver damage. Our data source does not contain adequate concomitant drug information to rule out the possible use of other hepatotoxic drugs. These effects are worthy of further investigation.

There are some limitations to this study, owing to the inherent drawbacks of real-world retrospective data (16). First, not all the results of each laboratory test were collected. The liver function tests were carried out on an irregular basis. Different patients might have undergone different numbers of tests throughout the hospitalization period. However, the use of growth curve analysis is an effective way to handle this complication (14). Second, the registry was not designed for this type of analysis. Many important variables were not collected. It is unknown how providers determined the basis of prescribing different types of treatments for each patient. Therefore, when we compare the effects of different treatments, the patient population might not be perfectly comparable. To address this issue, we included some major confounders, such as sex and age, in the analysis. In addition, the RWE serves as a supplement to an RCT. However, the control treatments mainly served as positive references. This is neither a superiority nor a non-inferiority study. The results of this study provide RWE, supporting the effectiveness of a PPC treatment in improving liver function in patients with CHB. Additionally, the database did not include data pertaining to the adverse events associated with the treatment. There was no planned long-term follow-up after 6 or 12 months. These issues need to be taken into consideration when designing a future prospective study.

## Conclusion

This was the first real-world study evaluating the effects of PPC on liver function in patients with CHB. Our results demonstrated that PPC treatment was effective in lowering ALT and AST levels in patients with and without CHB. Furthermore, high-dose PPC resulted in a faster decline in the levels of these proteins.

## Data Availability Statement

The original contributions presented in this study are included in the article/supplementary material, further inquiries can be directed to the corresponding author.

## Ethics Statement

The studies involving human participants were reviewed and approved by the Ethics Committee of Peking University First Hospital. The patients/participants provided their written informed consent to participate in this study.

## Author Contributions

JX, YF, and XX contributed to the study’s conception and design. All authors collected the data, performed the data analysis, and contributed to the interpretation of the data and the completion of figures and tables. All authors contributed to the drafting of the article and final approval of the submitted version.

## Conflict of Interest

The authors declare that the research was conducted in the absence of any commercial or financial relationships that could be construed as a potential conflict of interest.

## Publisher’s Note

All claims expressed in this article are solely those of the authors and do not necessarily represent those of their affiliated organizations, or those of the publisher, the editors and the reviewers. Any product that may be evaluated in this article, or claim that may be made by its manufacturer, is not guaranteed or endorsed by the publisher.
